# Effectiveness of Early Favipiravir Therapy in Hospitalised COVID-19 Patients

**DOI:** 10.1155/2022/9240941

**Published:** 2022-06-29

**Authors:** Abdulrahman Tawfik, Abdulrahman Alzahrani, Sami Alharbi, Jamal Almitairi, Arwa Alzahrani, Mohammed Ali Alshehri, Mohammed S. Aldughaim, Hani Alothaid

**Affiliations:** ^1^Medical Cluster of Infection Prevention and Control, Alqassim, Saudi Arabia; ^2^Department of Applied Medical Sciences, Applied College, Al Baha University, Al-Baha, Saudi Arabia; ^3^Department of Internal Medicine, King Fahad Specialist Hospital, Alqassim, Saudi Arabia; ^4^Department of Clinical Service Line, King Fahad Specialist Hospital, Alqassim, Saudi Arabia; ^5^College of Medicine, University of Hail, Saudi Arabia; ^6^Faculty of Applied Medical Sciences, Al Baha University, Al-Baha, Saudi Arabia; ^7^Research Center, King Fahad Medical City, P.O. Box. 59046, Riyadh 11525, Saudi Arabia

## Abstract

COVID-19 is a disease caused by a novel coronavirus with no specific, standard treatment. We investigated the clinical data of COVID-19 patients admitted to King Fahad Specialist Hospital (KFSH) in Buraydah by comparing the patients who were treated early with favipiravir (within 3 days of admission) to patients who were treated after three days of admission or not treated. 165 patients were confirmed with PCR tests and admitted to KFSH for treatment. Comorbidities contributed significantly to increasing the length of stay in hospital at 11.4 ± 0.8 days compared to patients with no comorbidities at 8.6 ± 0.9 days (*p*=0.041). A total of 103 patients were treated with favipiravir, and we found that early treatment with favipiravir (within 3 days) reduced the length of stay in hospital significantly (8.8 ± 1.4 days) compared to patients who were treated after 3 days (13.3 ± 4.6 days) (*p*=0.0015). Moreover, patients with comorbidities in both early and late treatment groups had significantly higher average lengths of stay in hospital (11.2 ± 0.9 days) compared to patients with no comorbidities (7.9 ± 0.7 days) (*p*=0.017). Interestingly, patients treated early with favipiravir (with comorbidities and without) stayed fewer days in hospital compared to those with late treatment (*p*=0.021; a difference of 4.5 ± 1.9 days; and *p*=0.018; a difference of 4.2 ± 1.7 days, respectively). In conclusion, our analysis indicates that early treatment with favipiravir can reduce the length of stay in hospital and improve clinical manifestations of COVID-19 patients.

## 1. Background

The ongoing global pandemic, known as COVID-19 disease, is caused by the severe acute respiratory syndrome coronavirus-2 (SARS-CoV-2), a single-stranded RNA virus from the family *Coronaviridae*, genus betacoronavirus, named SARS-CoV-2 [1]. Up until now, there has been continued increase in mortality due to COVID-19, with around four million deaths globally since it first appeared in China in late 2019. There is still no standard treatment for COVID-19, even though both SARS-CoV-2, MERS (Middle Eastern respiratory syndrome), and its related SARS-CoV are from the same family, the latter having a standard antiretroviral treatment such as lopinavir/ritonavir [2]. Antiviral treatment for MERS that is found to be beneficial for MERS and SARS-CoV patients is however known to not provide any beneficial effect on convalescence of patients with SARS-CoV-2 [2]. This is likely due to the genetic difference or mutations between these viral species.

While there is no cure for COVID-19, antiviral medication, such as favipiravir, can be metabolized into a nucleoside analogue, which inhibits RNA synthesis by the viral RNA-dependent RNA polymerase or RdRp [3]. Recently, favipiravir has been demonstrated to show efficacy in improving health outcomes of SARS-CoV-2 patients [4]. Other studies indicate specific useful application of early favipiravir treatment of COVID-19 patients in terms of reducing viral activity [5] and improving clinical signs in patients [6]. On this basis, the Ministry of Health in the Kingdom of Saudi Arabia (KSA) has recently proposed favipiravir as a treatment option for severe and hospitalised COVID-19 patients.

Based on the promise shown in earlier studies and its current use in KSA, this study examines the effectiveness of favipiravir treatment with severe COVID-19 patients in KSA in terms of length of hospital stay.

## 2. Materials and Methodology

### 2.1. Ethical Approval

The study was carried out at the King Fahad Specialist Hospital (KFSH) in Buraydah, Al Qassim Province, Saudi Arabia. The study was approved by the Public Health Research and Health Statistics (registration number 202010281, IRB number H-04-Q-001, date 04/10/2020), and it received ethics approval from Regional Research Ethics Committee, Al-Qassim Province, Saudi Arabia.

### 2.2. Patients

Patients with COVID-19 were admitted to the KFSH according to the following standard admission criteria of the Ministry of Health in Saudi Arabia: (1) clinical or radiological evidence of pneumonia; (2) low oxygen saturation (SpO2 < 94% on room air); (3) acute respiratory distress syndrome (ARDS); (4) chronic pulmonary disease; (5) chronic kidney disease; (6) history of comorbidities. *Diabetes mellitus* and/or hypertension; (7) history of cardiovascular disease; (8) obesity (BMI ≥40); (9) use of biological (immunosuppressant) medication (e.g., TNF inhibitors, interleukin inhibitors, and anti-B cell agents); (10) history of organ transplant or another immunosuppression disease; (11) history of active malignancy; (12) other coillnesses requiring admission [7].

This study used anonymised clinical data of patients following their discharge from the hospital. Because there was no access to patient's personal information, consent forms were not required. The study included 165 COVID-19 patients admitted to KFSH during the five-month period from 3 June to 3 November 2020. These COVID-19 patients had an average oxygen saturation (SpO2) of <90% and a respiratory rate of around 20/min and were either with or without comorbidities (Tables 1 and 2). Treatment with steroids (i.e., dexamethasone (6–12 mg once daily) or prednisolone (40 mg twice daily) and favipiravir (1800 mg dose per day on the first day, then 800 mg twice daily for 7–10 days) was standard for all positive PCR COVID-19 patients in this hospital [7]. However, due to the global demand for antiviral treatment (caused by the pandemic), there has been a shortage of favipiravir, which might explain the nonusage and late usage of the treatment with some patients. Therefore, this study used a Quasiexperimental design to compare favipiravir usage in confirmed COVID-19 patients (i.e., patients treated with favipiravir versus patients who were not treated with favipiravir).

### 2.3. Statistical Analysis

COVID-19 patient data collected from the KFSH were analyzed by GraphPad Prism 9.2.0 and an unpaired *T*-test (two-tailed) was carried out to compare each group. Clinical signs, such as fever, respiratory rate, and saturation (SpO2) were presented, as was the length of stay in hospital (days) and the mean length of stay in hospital between groups (± standard error of mean). Significant differences were presented as *p* value <0.05. Chi-square tests were also used to investigate the relationship between the probability of survival between those patients treated with favipiravir and those not treated with favipiravir.

## 3. Results

**Figure 1:**
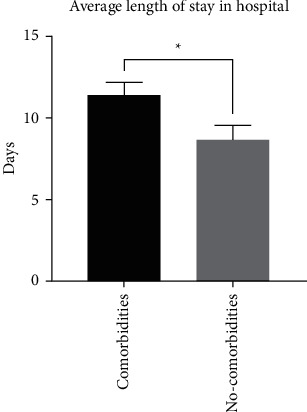
Average length of stay in hospital of COVID-19 patients with or without comorbidities in the total patient sample (treated with favipiravir or not) (*n* = 110, *n* = 51, respectively) (*p*=0.041).

This study examined data from 165 COVID-19 patients admitted to KFSH with a mean age of 60.1 ± 14.0 (Table 1). We observed that comorbidities contributed significantly to the length of stay in the hospital. COVID-19 patients with comorbidities stayed 11.4 ± 0.8 days (*n* = 110) compared to 8.6 ± 0.9 days (*n* = 51) for those with no comorbidities (*p*=0.041) (Figure 1).

A chi-square test was performed to examine the relationship between the probability of COVID-19 disease survival between the patients treated with favipiravir and untreated patients (patients not treated with favipiravir). Of the 103 COVID-19 patients treated with favipiravir, there was only one recorded mortality. However, there was 17 recorded mortalities in the group of 62 untreated patients. Interestingly, there was a significant difference in this recorded mortality between the two groups based on the chi-square test: X2 (1, *n* = 165) = 27.8564, *p* < 0.00001. This suggests that patients that were treated with favipiravir were more likely to survive COVID-19 disease compared with those who were not treated with favipiravir (Table 3).

**Figure 2:**
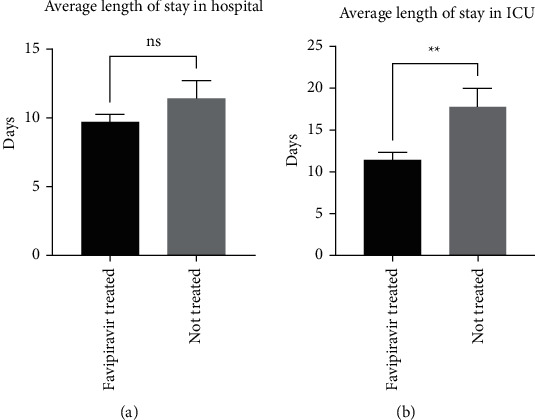
(a) Average length of stay in hospital for COVID-19 patients (favipiravir-treated and not treated with favipiravir) (*n* = 103, *n* = 62, respectively). (b) Average length of stay in ICU for COVID-19 patients (favipiravir-treated and not treated with favipiravir) (*n* = 21, *n* = 16, respectively) (*p*=0.0072).

Moreover, both groups of patients were admitted to the hospital for an average stay of 9.6 ± 1.2 days (*n* = 102) and 11.4 ± 1.7 days (*n* = 58), respectively. Whilst there was a trend of fewer days spent in hospital among the favipiravir-treated COVID-19 patients, the difference was not significant (Figure 2(a)). However, there was significant difference in the length of days stayed in the intensive care unit (ICU) by some of these favipiravir-treated and nonfavipiravir-treated patients (11.4 ± 6.3 days (*n* = 21) and 17.7 ± 2.2 days (*n* = 16), respectively, *p*=0.0072) (Figure 2(b)).

**Figure 3:**
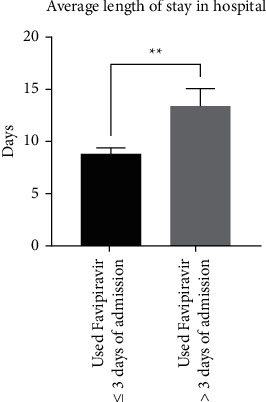
Comparison between patients treated with favipiravir within three days of admission (*n* = 75) or more than three days after admission (*n* = 28) in terms of the average length of stay in hospital (*p*=0.0015).

Next, we divided our COVID-19 patients who had received favipiravir into two categories: patients treated within three days of admission (≤3 days) and patients treated more than three days after admission (>3 days), based on previous studies [5,8,9] (Table 2). Convalescence for patients treated within ≤3 days of admission was significantly reduced (8.8 ± 1.4 days; *n* = 75) compared to patients who were treated >3 days after admission (13.3 ± 4.6 days; *n* = 28) (*p* = 0.0015) (Table 2 and Figure 3).

**Figure 4:**
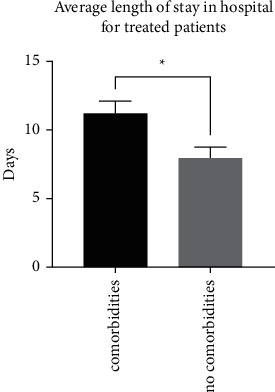
Comparison between patients with comorbidities (*n* = 66) and without comorbidities (*n* = 37) in terms of the average length of stay in hospital (both groups were treated with favipiravir) (*p*=0.017).

**Figure 5:**
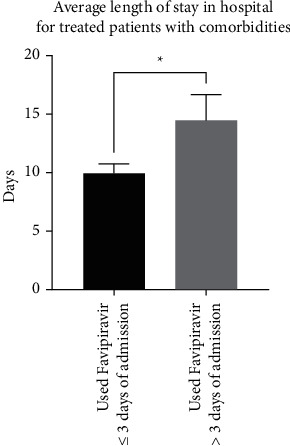
Comparison between patients with comorbidities who were treated with favipiravir within 3 days of admission (*n* = 47) and more than 3 days after admission (*n* = 19) in terms of the average length of stay in hospital (*p*=0.021).

**Figure 6:**
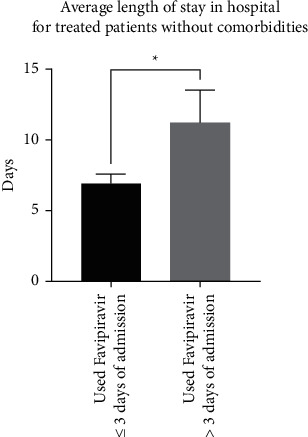
Comparison between patients without comorbidities who were treated with favipiravir within three days of admission (*n* = 47) and patients treated more than three days after admission (*n* = 19) in terms of the average length of stay in hospital (*p*=0.018).

Similar to the previous finding, patients with comorbidities in both categories of treatment had significantly higher average lengths of stay in hospital (11.2 ± 0.9 days; *n* = 66) compared to patients with no comorbidities (7.9 ± 0.7 days; *n* = 37) (*p*=0.017; a difference of 3.2 ± 1.3 days) (Figure 4). Interestingly, patients with comorbidities who were treated within three days of admission stayed significantly fewer days in the hospital, with an average of 9.9 ± 0.8 days (*n* = 47) compared to patients treated >3 days after admission (14.4 ± 2.1 days; *n* = 19) (*p*=0.021; a difference of 4.5 ± 1.9 days) (Figure 5). Moreover, patients without comorbidities who were treated within three days of admission stayed significantly fewer days in hospital, with an average of 6.9 ± 0.6 days (*n* = 28) compared to patients treated >3 days after admission (11.2 ± 2.3 days; *n* = 9) (*p*=0.018; a difference of 4.2 ± 1.7 days) (Figure 6).

**Figure 7:**
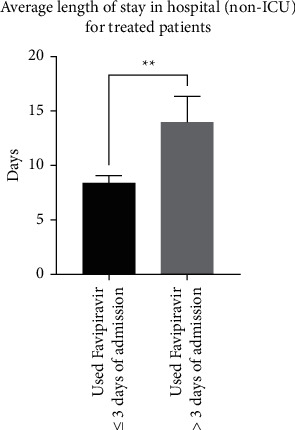
Comparison between patients in hospital (non-ICU) treated with favipiravir within 3 days of admission (*n* = 12) or after 3 days of admission (*n* = 9) in terms of the average length of stay in hospital (*p*=0.0025).

**Figure 8:**
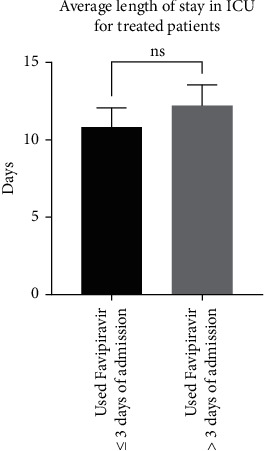
Comparison between patients in the ICU treated with favipiravir within 3 days of admission (*n* = 63) or after 3 days of admission (*n* = 19) in terms of the average length of stay in hospital (*p* > 0.05).

Interestingly, we observed a similar trend in patients that were not transferred to the ICU, with an average length of stay in hospital of 8.4 ± 0.6 days (*n* = 63) for patients treated within ≤3 days compared to 14 ± 2.3 days (*n* = 19) for patients treated >3 days after admission (*p*=0025; a difference of 8.4 ± 1.7 days) (Figure 7). In terms of patients who were transferred to the ICU, there was a trend (but not statistically significant) of an average stay at hospital of 10.3 ± 1.2 days (*n* = 12) and 12.2 ± 1.3 days (*n* = 9) for patients treated within <3 days compared to patients treated >3 days after admission, respectively (Figure 8).

## 4. Discussion

Globally, age and comorbidities are risk factors for convalescence, mortality, and length of stay in hospital for patients with COVID-19 [10,11]. In our results, we observed the same outcome, suggesting that age and comorbidities are clinical risk factors of mortality and should be taken into consideration in determining the need for COVID-19 therapy. Interestingly, a study in 2020 deduced that comorbidities were the main cause of clinical complications in COVID-19 patients [12], suggesting the importance of considering this risk factor for patient treatment.

At present, no standard treatment has been applied for all COVID-19 patients, but current interventions include steroids, antimicrobial drugs, antiviral (favipiravir) drugs, or oxygen supplements. KSA, Russia, India, and Turkey have now approved the use of favipiravir with COVID-19 patients due to its ability to reduce symptoms of COVID-19 infection [4,13]. Indeed, favipiravir has shown a marked effect in COVID-19 patients, reducing fever and the presence of coughs [14]. The results of this study agree with those of previous studies in that we found that favipiravir increased the survival rate and decreased the length of stay in hospital for COVID-19 patients. Furthermore, another clinical study has shown favipiravir demonstrated clinical efficacy in the treatment of COVID-19 patients with minimal side effects [6]. Another study also showed that favipiravir compared with treatment with lopinavir/ritonavir produced significantly shorter median length of time to viral clearance as well as improvement in the chest CT scan 14 days after randomization with lower incidence of adverse effects [9].

Another point to note is that studies have shown that the level of viremia and the time of antiviral treatment correlate negatively with the clinical manifestations and mortality of COVID-19 patients [5,15]. In our analysis, we showed that early treatment with favipiravir (within three days of admission) resulted in patients being discharged from the hospital earlier and caused faster clinical improvement in terms of fever, respiratory rate, and saturation (SpO2) compared to late treatment (<3 days after admission). Other studies support this finding, verifying that the viral load in infected COVID-19 patients increases in the respiratory tract within four days [15], and this can be prevented by antiviral treatment (arbidol, interferon, oseltamivir, ribavirin, and ganciclovir) [9]. Indeed, Yu et al. [9] indicated that viral clearance was faster in COVID-19 patients who received antiviral treatment earlier when compared with the group treated later, with a seven-day difference between groups [9]. It should be noted that, due to the strain on the health care system during this pandemic, we could not investigate the viral clearance in any of our COVID-19 patients, those with early, late, and no favipiravir treatment.

The goal of this study was to observe the clinical improvement of favipiravir-treated COVID-19 patients in terms of high fever, low respiratory rate, and low saturation (SpO_2_), with the clinical parameter being the discharge day from the hospital. In 2020, Ting Yu and Heng Fan showed that treating COVID-19 patients at disease onset with different antivirals, such as arbidol, ribavirin, and oseltamivir, improved their symptoms within six days [9]. Karatas et al. [5] reported similar results. Their study indicated that the need for respiratory support (including supplemental oxygen, high-flow nasal oxygen, and invasive mechanical ventilation) for patients with COVID-19 treated with favipiravir within 72 hours of hospital admission reduced significantly compared to the late treatment group [5]. A similar observation was made in our study where COVID-19 patients who were treated within three days with favipiravir compared to those patients who were treated more than three days after admission.

The chances of rapid duplication of COVID-19 in the body over time is well known. This viremia and virus replication results in serious symptoms, one of which is the triggering of cytokine storms, including interferon-alpha, IL-2, IL-6, IL-10, and C-reactive protein, which can erode the health of COVID-19 patients [16]. An early intervention treatment with an antiviral can reduce the rate of virus replication [3] and thus reduce the immunological cytokine storm. In a similar vein, a study in 2020 by Doi et al. found that administering favipiravir to COVID-19 patients reduced fever within a day [8]. Therefore, favipiravir treatment within three days for COVID-19 patients shortens the length of stay in the hospital compared to COVID-19 patients treated more than three days after admission by inhibiting the cause of the disease.

It is known that comorbidities can speed up the progression of COVID-19 infection [12]. In our study, COVID-19 patients with comorbidities had a longer stay in the hospital compared to patients with no comorbidities. This was similar in patients transferred to the ICU because of other complications that occur with COVID-19 infection. Indeed, certain comorbidities such as cardiovascular disease, diabetes, and chronic respiratory disease may worsen the COVID-19 infection [17]. That said, there has been no clear indication of how chronic diseases could worsen the progression of COVID-19 infection, yet, treatment with favipiravir within three days appears to shorten the length of stay in the hospital of COVID-19 sufferers.

In conclusion, we have found a strong relationship between early treatment of COVID-19 patients with favipiravir (within three days) and clinical improvement and reduced mortality in COVID-19 patients. This was observed in all treated patients (those with and without comorbidities). In our study, we refer to the length of stay in the hospital as an indication of the need for clinical attention to fever, respiratory rate, and saturation (SpO_2_), as these are signs of clinical complications and mortality. The limitations of this study were that it has not examined clinical laboratory parameters (including white blood cells, haemoglobin, and platelets) before and after patients were discharged from the hospital. We also did not investigate the viral load after treatment. The latter was due to the high demands on the hospital at the time of admission, and thus the data being unavailable.

## Figures and Tables

**Table 1:** Patients' raw data.

	Treated with favipiravir (*n* = 103)	Not treated (*n* = 62)	
Ages, mean (SEM)	58.2 (1.6)	63.3 (2.2)	
Survival/death (%)	102/1 (99.1%–0.9%)	45/17 (72.6%–27.4%)	*P*=0.0002^*∗*^
Non-ICU/ICU (%)	82/21 (79.6%–20.4%)	46/16 (74.2%–25.8%)	
Comorbidities/no comorbidities (%)	66/37 (64.1%–35.9%)	48/14 (77.4%–22.6%)	
Days in hospital (SEM)	9.6 (0.5)	11.4 (1.2)	

^*∗*^Difference between the mean age of survival and death rate of COVID-19 patients (*n* = 165).

**Table 2:** Patients treated with favipiravir.

Treated with favipiravir	Within three days of admission (*n* = 75)	More than three days after admission (*n* = 28)	
Ages, mean (SEM)	57.8 (1.8)	59.2 (3.4)	
Survival/death (*n*) (%)	75/0 (100%–0%)	27/1 (96.4%–3.6%)	
Non-ICU (*n*) (%)	63 (84%)	19 (67.9%)	
ICU (*n*) (%)	12 (16%)	9 (32.1%)	
Comorbidities/no comorbidities (*n*) (%)	47/28 (62.7%–37.3%)	19/9 (67.9%–32.1%)	
Days in hospital (SEM)	8.8 (0.6)	13.4 (1.6)	*p*=0.0015

**Table 3:** Probability of survival of COVID-19 patients (with and without favipiravir treatment).

	Death	Survival	Marginal row totals
Treated with favipiravir	1 (11.24) [9.33]	102 (91.76) [1.14]	103
Not treated	17 (6.76) [15.49]	45 (55.24) [1.9]	62
Marginal column totals	18	147	165 (grand total)

The chi-square statistic is 27.8564. The *p* value is  < 0.00001.

## Data Availability

The data used to support this study are available from the corresponding author upon request.

## References

[1] Helmy Y. A., Fawzy M., Elaswad A., Sobieh A., Kenney S. P., Shehata A. A. (2020). The COVID-19 pandemic: a comprehensive review of taxonomy, genetics, epidemiology, diagnosis, treatment, and control. *Journal of Clinical Medicine*.

[2] Ford N., Vitoria M., Rangaraj A., Norris S. L., Calmy A., Doherty M. (2020). Systematic review of the efficacy and safety of antiretroviral drugs against SARS, MERS or COVID-19: initial assessment. *Journal of the International AIDS Society*.

[3] Furuta Y., Takahashi K., Shiraki K. (2009). T-705 (favipiravir) and related compounds: novel broad-spectrum inhibitors of RNA viral infections. *Antiviral Research*.

[4] Joshi S., Parkar J., Ansari A. (2021). Role of favipiravir in the treatment of COVID-19. *International Journal of Infectious Diseases: International Journal of Infectious Diseases*.

[5] Karatas E., Aksoy L., Ozaslan E. (2021). Association of early favipiravir use with reduced COVID-19 fatality among hospitalized patients. *Infection and Chemotherapy*.

[6] Cai Q., Yang M., Liu D. (2020). Experimental treatment with favipiravir for COVID-19: an open-label control study. *Engineering Times*.

[7] Ministry of Health (2020). Saudi MoH protocol for patients suspected of/confirmed with COVID-19. https://www.moh.gov.sa/Ministry/MediaCenter/Publications/Documents/MOH-therapeutic-protocol-for-COVID-19.pdf.

[8] Doi Y., Hibino M., Hase R. (2020). A prospective, randomized, open-label trial of early versus late favipiravir therapy in hospitalized patients with COVID-19. *Antimicrobial Agents and Chemotherapy*.

[9] Yanai H. (2020). Favipiravir: a possible pharmaceutical treatment for COVID-19. *Journal of Endocrinology and Metabolism*.

[10] Biswas M., Rahaman S., Biswas T. K., Haque Z., Ibrahim B. (2021). Association of sex, age, and comorbidities with mortality in COVID-19 patients: a systematic review and meta-analysis. *Intervirology*.

[11] Bonanad C., García-Blas S., Tarazona-Santabalbina F. (2020). The effect of age on mortality in patients with COVID-19: a meta-analysis with 611,583 subjects. *Journal of the American Medical Directors Association*.

[12] Wu J., Li W., Shi X. (2020). Early antiviral treatment contributes to alleviate the severity and improve the prognosis of patients with novel coronavirus disease (COVID‐19). *Journal of Internal Medicine*.

[13] Wang M., Cao R., Zhang L. (2020). Remdesivir and chloroquine effectively inhibit the recently emerged novel coronavirus (2019-nCoV) in vitro. *Cell Research*.

[14] Chen C., Zhang Y., Huang J. (2021). Favipiravir versus arbidol for clinical recovery rate in moderate and severe adult COVID-19 patients: a prospective, multicenter, open-label, randomized controlled clinical trial. *Frontiers in Pharmacology*.

[15] Magleby R., Westblade L. F., Trzebucki A. (2020). Impact of SARS-CoV-2 viral load on risk of intubation and mortality among hospitalized patients with coronavirus disease 2019. *Clinical Infectious Diseases*.

[16] Han H., Ma Q., Li C. (2020). Profiling serum cytokines in COVID-19 patients reveals IL-6 and IL-10 are disease severity predictors. *Emerging Microbes & Infections*.

[17] Wu Z., McGoogan J. M. (2020). Characteristics of and important lessons from the coronavirus disease 2019 (COVID-19) outbreak in China. *Journal of the American Medical Association*.

